# Deployment and assessment of a deep learning model for real-time detection of anal precancer with high frame rate high-resolution microendoscopy

**DOI:** 10.1038/s41598-023-49197-9

**Published:** 2023-12-14

**Authors:** David Brenes, Alex Kortum, Jackson Coole, Jennifer Carns, Richard Schwarz, Imran Vohra, Rebecca Richards-Kortum, Yuxin Liu, Zhenjian Cai, Keith Sigel, Sharmila Anandasabapathy, Michael Gaisa, Elizabeth Chiao

**Affiliations:** 1 Department of Bioengineering, Rice University, MS-142 6100 Main St., Houston, TX 77005 USA; 2 Biotex, 114 Holmes Rd., Houston, TX 77045 USA; 3 Department of Pathology, Icahn School of Medicine at Mount Sinai, 1468 Madison Avenue, New York, NY 10029 USA; 4 Clinical Pathology Laboratories, 9200 Wall Street, Austin, TX 78754 USA; 5 Department of Medicine, Icahn School of Medicine at Mount Sinai, One Gustave Levy Place, New York, NY 10029 USA; 6 Department of Medicine, Baylor College of Medicine, One Baylor Plaza, Houston, TX 77030 USA; 7 Department of Epidemiology, Division of Cancer Prevention, University of Texas – MD Anderson Cancer Center, 1155 Pressler St., Unit 1340, Houston, TX 77030 USA; 8 Department Epidemiology, Division of Cancer Prevention, and Department of General Oncology, Division of Cancer Medicine, University of Texas – MD Anderson Cancer Center, 1155 Pressler St., Unit 1340, Houston, TX 77030 USA

**Keywords:** Cancer imaging, Cancer prevention, Anal cancer, Diagnostic markers

## Abstract

Anal cancer incidence is significantly higher in people living with HIV as HIV increases the oncogenic potential of human papillomavirus. The incidence of anal cancer in the United States has recently increased, with diagnosis and treatment hampered by high loss-to-follow-up rates. Novel methods for the automated, real-time diagnosis of AIN 2+ could enable "see and treat" strategies, reducing loss-to-follow-up rates. A previous retrospective study demonstrated that the accuracy of a high-resolution microendoscope (HRME) coupled with a deep learning model was comparable to expert clinical impression for diagnosis of AIN 2+ (sensitivity 0.92 [P = 0.68] and specificity 0.60 [P = 0.48]). However, motion artifacts and noise led to many images failing quality control (17%). Here, we present a high frame rate HRME (HF-HRME) with improved image quality, deployed in the clinic alongside a deep learning model and evaluated prospectively for detection of AIN 2+ in real-time. The HF-HRME reduced the fraction of images failing quality control to 4.6% by employing a high frame rate camera that enhances contrast and limits motion artifacts. The HF-HRME outperformed the previous HRME (P < 0.001) and clinical impression (P < 0.0001) in the detection of histopathologically confirmed AIN 2+ with a sensitivity of 0.91 and specificity of 0.87.

## Introduction

The incidence rate of anal cancer is more than 80 times higher in people living with human immunodeficiency virus (PLWH) than in the uninfected population^1^. PLWH are at a higher risk of coinfection with human papillomavirus (HPV), which has been linked to 90% of anal cancer cases^2^. HPV vaccination is highly effective as a primary prevention strategy against new HPV infection^3^. However, secondary prevention strategies remain necessary to prevent disease for PLWH who are unvaccinated or have a history of persistent anal HPV or anal precancer^4^.

In the United States, secondary prevention begins with screening high-risk individuals using anal cytology^5^. PLWH, whose anal cytology yields atypical squamous cells of undetermined significance (ASCUS) or higher-grade cellular abnormalities, are referred for high-resolution anoscopy (HRA), a procedure involving optically-assisted visual inspection of the anal canal using a colposcope. During HRA, biopsies are taken from any suspicious lesions and sent for histopathologic diagnosis. Possible histopathologic diagnoses include benign, anal intraepithelial neoplasia (AIN) 1, condyloma acuminatum, AIN 2, AIN 3, or cancer, in order of increasing severity. Patients with a histopathologic diagnosis of AIN 2 or more severe (AIN 2+) must return for treatment at a subsequent visit. While treating anal high-grade squamous intraepithelial lesions (HSIL) has been shown to prevent incident anal cancer successfully, the limited availability of trained HRA practitioners and a high patient loss-to-follow-up rate challenge the widespread implementation of these strategies^6–9^. Consequently, there is an urgent need for imaging techniques that can provide a real-time, automated diagnosis to expedite treatment and expand access to care.

To assist novice HRA practitioners and potentially enable same-day treatment, Brenes et al. evaluated the performance of an in vivo imaging system for automated detection of anal precancer^10^. In this pilot study, the anal epithelium was imaged with sub-cellular resolution using a high-resolution microendoscope (HRME) operating at 15 frames per second; a multi-task neural network (MTN) was used to predict pathology from acquired HRME images. In a retrospective assessment, the accuracy of HRME was comparable to expert HRA impression for diagnosis of AIN 2+ (sensitivity of 0.92 [P = 0.68] and specificity 0.60 [P = 0.48]) using histopathology as the gold standard. However, a high fraction of images (17%) failed manual quality control, which could impact clinical deployment^10^. Poor image quality was associated with motion blur and poor tissue contact, a particular challenge given the complex anatomy of the anal canal and the difficulty of holding the handheld HRME probe steady during image acquisition.

In this work, we sought to evaluate whether increasing the frame rate of the HRME system could improve image quality and diagnostic performance. We used a high frame rate HRME (HF-HRME) system, incorporating a high frame rate camera, to enhance image contrast, reduce motion blur, and allow for faster probe translation during in vivo imaging. Compared to the pilot study of the original HRME, the fraction of images that passed quality control increased from 83% with the original HRME to 95% with the HF-HRME. Diagnostic accuracy also improved; the HF-HRME outperformed the original HRME with a specificity of 0.87 (P < 0.001) at a comparable sensitivity of 0.91 (P = 1.0). An image perturbation study was performed, and results show that both improved image contrast and motion blur reduction contributed significantly to the improved diagnostic performance.

## Results

### Data collection and diagnostic performance

**Figure 1:**
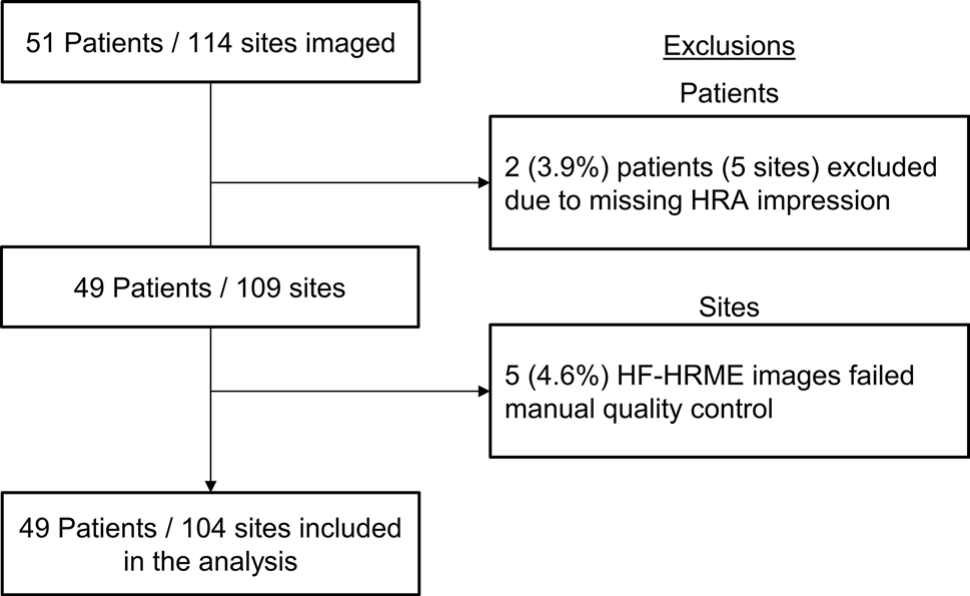
Flow diagram detailing subject enrollment and exclusions applied to generate the analysis set. *HF-HRME* high frame rate high-resolution microendoscope, *HRA* high-resolution anoscopy.

**Figure 2:**
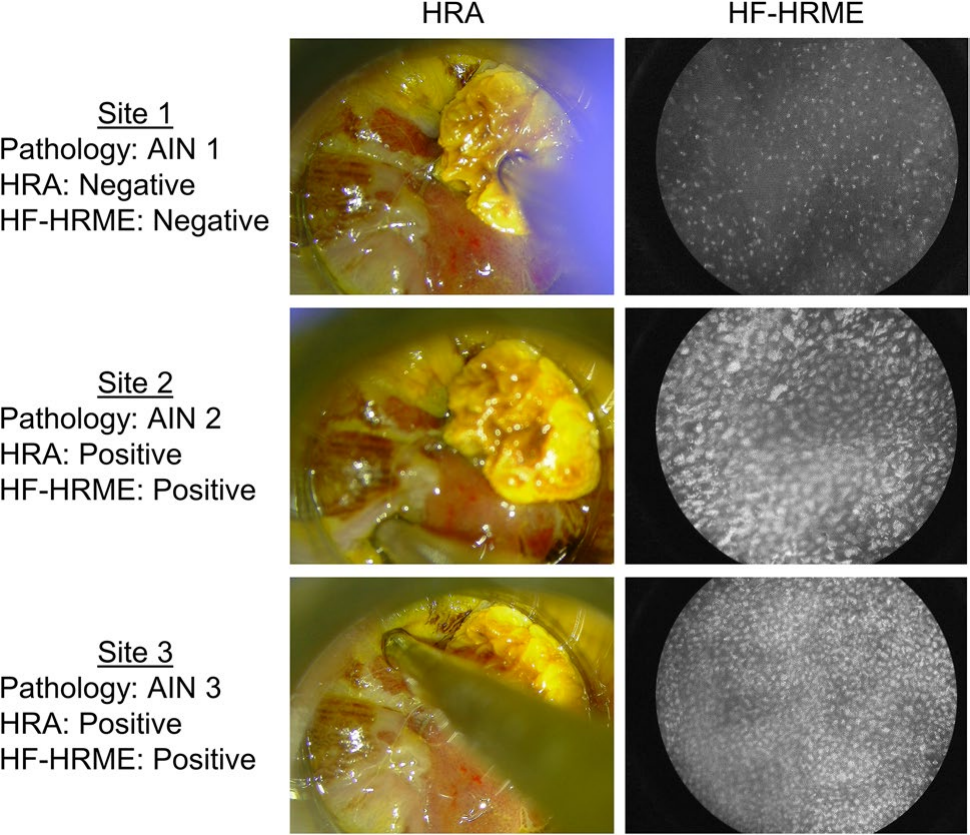
Representative HF-HRME images acquired from three sites selected for biopsy by HRA impression. Site 1 was an area with histologically confirmed AIN 1, HF-HRME score 0.08 (HF-HRME negative), and negative HRA impression. Site 2 was an area with histologically confirmed AIN 2, HF-HRME score 0.67 (HF-HRME positive), and positive HRA impression. Site 3 was an area with histologically confirmed AIN 3, HF-HRME score 0.71 (HF-HRME positive), and positive HRA impression. Refer to Supplemental Video 1 for a video of the imaging session. *HF-HRME* high frame rate high-resolution microendoscope, *HRA* high-resolution anoscopy, *AIN 1* anal intraepithelial neoplasia grade 1, *AIN 2* anal intraepithelial neoplasia grade 2, *AIN 3* anal intraepithelial neoplasia grade 3.

This study enrolled a total of 51 PLWH. Two patients missing HRA impressions were excluded from the analysis set. Images were collected from 109 sites in the remaining 49 patients; images from 4.6% of sites failed manual quality control (Fig. 1). The analysis set consists of images from 104 sites from 49 patients with correlated histopathologic diagnoses, HRA impressions, and real-time MTN scores. Table 1 summarizes the number of sites by histopathologic diagnosis. The per site AIN 2+ prevalence was 21%. Figure 2 shows an example of the data collected from three biopsy sites selected by HRA impression for one patient (Supplemental Video 1). Site 1 with AIN 1 had an HF-HRME score of 0.08 (HF-HRME negative) and a negative HRA impression. Site 2 with AIN 2 and Site 3 with AIN 3 had HF-HRME scores of 0.67 and 0.71, respectively; both sites were positive by HF-HRME and HRA impression.

**Table 1:** Histopathologic diagnoses for sites in the analysis set imaged with HF-HRME.

Histopathologic diagnosis	Number of sites
Benign	11 (11%)
AIN 1	71 (68%)
AIN 2+	22 (21%)
Total	104 (100%)

*HF-HRME* high frame rate high-resolution microendoscope, *AIN 1* anal intraepithelial neoplasia grade 1, *AIN 2+ *anal intraepithelial neoplasia grade 2 or more severe.

**Figure 3:**
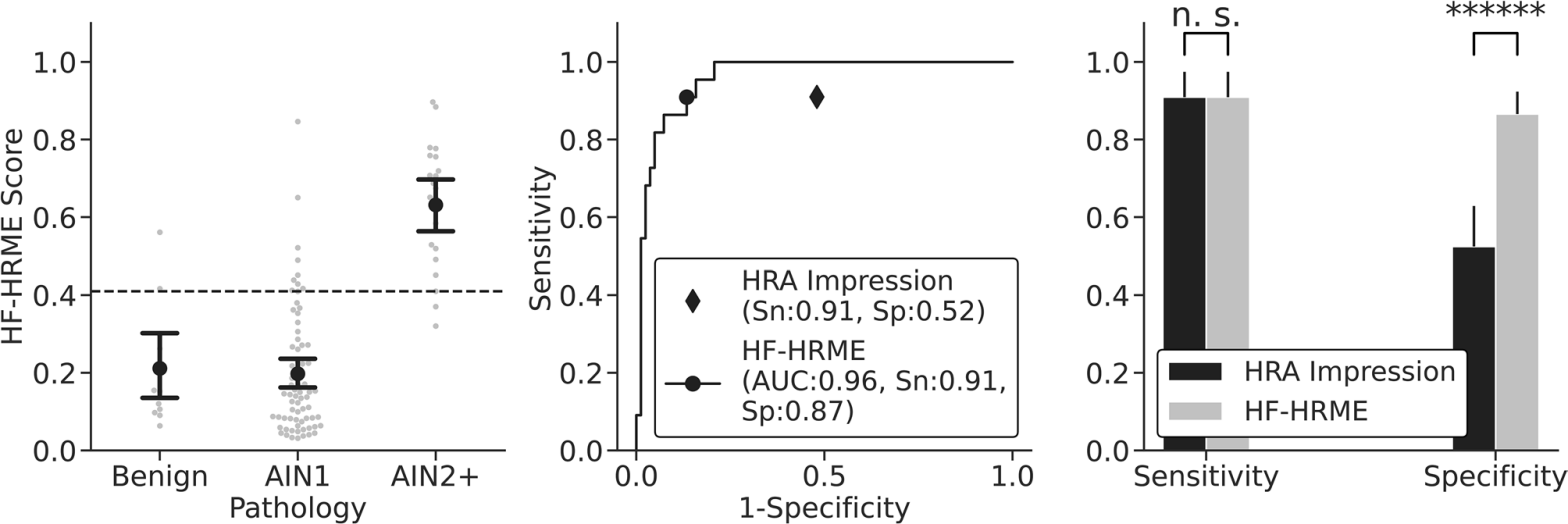
Diagnostic performance of HF-HRME with MTN analysis compared to HRA impression using histopathology as the gold standard. (**a**) Per site probability of AIN 2+ from HF-HRME images stratified by histopathologic diagnosis. Histopathologic diagnosis of AIN 2+ was considered positive. Error bars indicate the mean and 95% confidence interval, while the solid line across all classes denotes a retrospective cutoff to discriminate AIN 2+ lesions. (**b**) The receiver operating characteristic curve for HF-HRME with MTN analysis; operating points for HF-HRME and HRA impression are indicated with symbols. (**c**) Sensitivity and specificity of HF-HRME and HRA impression at operating points with similar sensitivity. Error bars indicate 95% confidence intervals. *HF-HRME* high frame rate high-resolution microendoscope, *MTN* multi-task network, *AIN 1* anal intraepithelial neoplasia grade 1, *AIN 2+ *anal intraepithelial neoplasia grade 2 or more severe, *AUC* area under the receiver operating characteristic curve, *Sn* sensitivity, *Sp* Specificity, *HRA* high-resolution anoscopy.

Figure 3 shows the prospective, real-time diagnostic performance of the MTN. Figure 3a shows the AIN 2+ probabilities of all sites in the analysis set stratified by histopathologic diagnosis with error bars indicating the mean and 95% confidence intervals. The cutoff threshold for HF-HRME positivity was 0.4. Figure 3b shows the receiver operating characteristic (ROC) curve of the HF-HRME; the area under the ROC was 0.96, an 11.6% increase compared to that reported in the original pilot study^10^. The area under the precision-recall curve was 0.83. Figure 3b also shows the sensitivity and specificity of HRA impression and the operating point for HF-HRME with equivalent sensitivity to HRA impression. At this operating point, HF-HRME had a comparable sensitivity to HRA impression of 0.91 (P = 1.0, 95% CI 0.72–0.97) but had a higher specificity of 0.87 (P < 0.0001, 0.77–0.92) (Fig. 3c). Table 2 shows the agreement between HF-HRME prediction and HRA impression stratified by histopathologic diagnosis. The per site diagnoses for HF-HRME and HRA impression agreed for 68 of the 104 sites (65%) (κ = 0.34). Of the 36 discordant sites, the majority (28 of 36 discordant sites; 78%) were histologically confirmed as AIN 1, correctly identified as negative by HF-HRME, and incorrectly identified as positive by HRA impression. The HF-HRME outperformed the original HRME with a specificity of 0.87 (P < 0.001) at a comparable sensitivity of 0.91 (P = 1.0).

**Table 2:** Agreement between HF-HRME prediction and HRA impression stratified by histopathologic diagnosis.

HF-HRME	HRA	Histopathologic diagnosis		
		Benign (n = 11)	AIN 1 (n = 71)	AIN 2+ (n = 22)
–	–	7	34	0
–	+	2	28	2
+	–	0	2	2
+	+	2	7	18

*HF-HRME* high frame rate high-resolution microendoscope, *HRA* high-resolution anoscopy, *AIN 1* anal intraepithelial neoplasia grade 1, *AIN 2+ *anal intraepithelial neoplasia grade 2 or more severe.

The differences in contrast and noise floor between the HF-HRME and the original HRME were evaluated. The HF-HRME had a Weber fraction of 7.3, while the original HRME had a Weber fraction of 1.7. Furthermore, imaging of a blank target showed that the noise floor of the original HRME was four times higher than the noise floor of the HF-HRME.

### Image perturbation analysis

**Figure 4:**
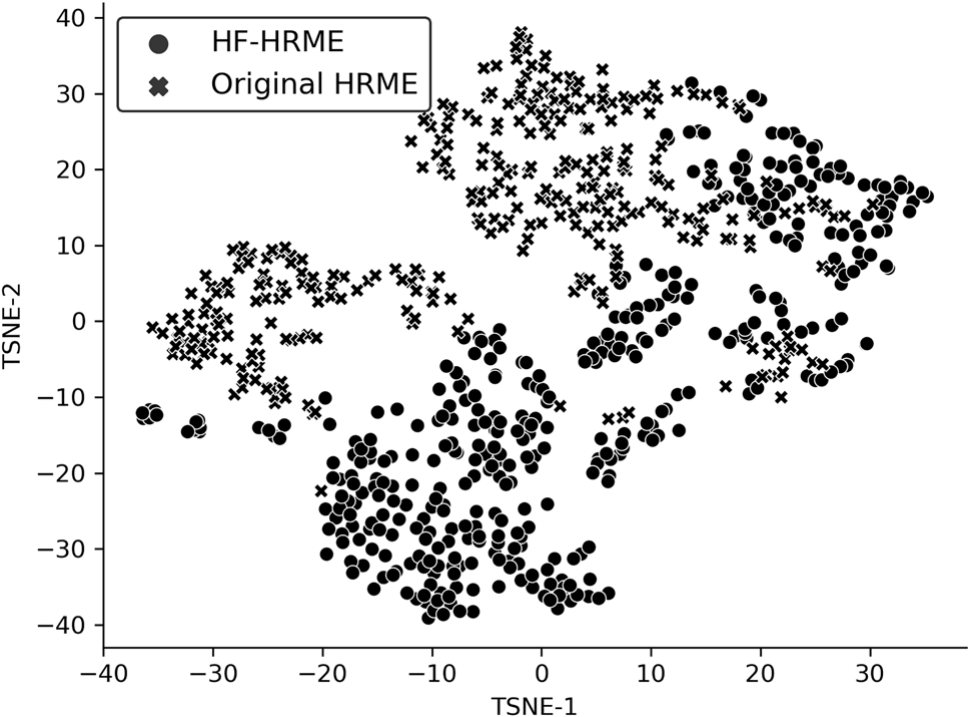
t-SNE visualization of features generated by the MTN from images acquired with HF-HRME in the current study and with the original HRME in the pilot study. Each point corresponds to features from a single image quadrant and is labeled by study. Study-specific clusters indicate a significant feature shift. *t-SNE* t-distributed stochastic neighbor embedding, *MTN* multi-task network, *HF-HRME* high frame rate high-resolution microendoscope, *HRME* high-resolution microendoscope.

Figure 4 illustrates the shift in MTN features observed between images acquired in this study with the HF-HRME and images acquired in the original pilot study with the HRME. Study-specific clusters indicate a significant feature shift. The Kullback–Leibler (K–L) divergence between HF-HRME and HRME image features was 0.433. See Supplemental Fig. 1 for a visualization of the clusters labeled by histopathologic diagnosis.

HF-HRME images acquired in this study were transformed to simulate the reduction in frame rate and contrast observed in the HRME data in the original study. Table 3 shows how these transformations lowered K–L divergence between the HF-HRME and HRME datasets. Method 1 applied all proposed transformations (gamma correction, fiber core smoothing, noise injection, saturation preservation, and contrast reduction) to the HF-HRME images and decreased the K–L divergence to 0.211. Methods 2 to 6 were part of the ablation study and showed that the K–L divergence increased when the contrast reduction or fiber core smoothing transformations were not applied.

**Table 3:** Impact of the transformation pipeline on K–L divergence and area under the receiver operating curve (AUC).

Method	Simulation methods					Assessment metrics	
	Blur synthesis module				Contrast reduction module		
	Gamma correction	Fiber core smoothing	Noise injection	Saturation preservation		K–L divergence	AUC
1	+	+	+	+	+	0.211	0.87
2	−	+	+	+	+	0.186	0.87
3	+	−	+	+	+	0.248	0.88
4	+	+	−	+	+	0.188	0.88
5	+	+	+	−	+	0.201	0.85
6	+	+	+	+	−	0.376	0.95
Original dataset	−	−	−	−	−	0.433	0.96

A positive sign (+) indicates when a module was present, and a negative sign (−) indicates when a module was absent. *K–L divergence* Kullback–Leibler divergence, *AUC* area under the receiver operating characteristic curve.

The lowest K–L divergence (0.186) was achieved using Method 2, where all proposed transformations were applied except the gamma correction. Method 2 was selected for further evaluation. A qualitative comparison between HF-HRME images, corresponding images transformed with Method 2, and representative images collected in the original pilot study is shown in Supplemental Fig. 2. When the ROC curve was calculated for HF-HRME data processed using Method 2, the area under the ROC curve (AUC) decreased from 0.96 to 0.87. This decrease in AUC was driven by an increase in the scores of sites diagnosed as AIN 1 (average increase of 90%) and a decrease in the scores of sites diagnosed as AIN 2+ (average decrease of 35%). We compared the MTN scores of the HF-HRME data transformed with Method 2 to the scores for data in the original pilot study; scores were stratified by histopathologic diagnosis for comparison. No statistically significant differences were found for benign (P = 0.84), AIN 1 (P = 0.63), and AIN 2+ (P = 0.15) sites.

## Discussion

The results of the Anal Cancer/HSIL Outcomes Research study by Lee et al. showed that treatment of anal precancers effectively prevents progression^9^. This result reaffirms the need for technologies to effectively and rapidly diagnose anal precancer. Non-invasive optical imaging systems like the HF-HRME have the potential to enable “see and treat” strategies for anal precancer prevention that may reduce the patient loss-to-follow-up rate and improve patient outcomes.

In this study, the HF-HRME outperformed both HRA impression and the performance previously reported for the original HRME system by providing higher specificity at comparable sensitivity. Our image perturbation analysis suggests that the superior diagnostic performance of the HF-HRME with respect to the original HRME is largely due to a reduction in motion blur and an increase in image contrast. Still, the image perturbation study could not eliminate the K–L divergence between the transformed HF-HRME data and the original pilot study data, suggesting that differences between feature distributions remain, albeit to a smaller extent. These differences may be associated with other instrumentation changes not captured by the transformation pipeline or clinical factors such as patient population and user variations.

A shift in predicted feature distributions has often been associated with a degradation in the predictive performance of the deep learning model^11^. Here, we observed the opposite, a shift in feature distributions that improved predictive performance. While we expected the higher image quality of the HF-HRME to improve diagnostic feature extraction, we anticipated that the MTN would have to be retrained on HF-HRME data before we could observe an improvement in diagnostic performance. The fact that no retraining was necessary suggests that the MTN has some inherent robustness to changes in acquisition parameters, a positive attribute for clinical deployment.

The MTN was trained for cervical precancer detection and was applied, without retraining, for anal precancer detection. There is a large body of literature documenting the similarities between anal and cervical precance^12,13^. Both anal and cervical precancers are HPV-associated squamous lesions. While there are some key differences in their epidemiology, progression risk, and treatment response, histologically the similarities between lesions overshadow any differences^14^. Since the HRME captures the underlying histology of the lesion, we anticipated that a model trained for cervical precancer detection could perform well in anal precancer detection. Due to the scarcity of AIN HRME data, it is not feasible to develop a robust MTN model solely using AIN data, as previous research indicates that more than 900 HRME images are needed for training^15^. In addition to our work, others have also exploited the similarities between cervical and anal precancers in the development of deep learning models^16^.

Our results suggest that HRME systems may benefit from continued improvements in contrast and new image quality assessment methods. Previous efforts to improve HRME instrumentation have sought to stabilize the fiber-optic probe and increase nuclear contrast. The flexibility and small diameter of a bare fiber-optic probe make it difficult for a user to maintain precise control as the probe is translated across the tissue surface during an imaging session. Lack of control can result in out-of-contact frames and rapid, sudden movements that contribute to a blurry image. To address this challenge, a tapered plastic probe holder was designed that provides a thicker handle and allows the clinician to firmly grip the probe. This holder significantly improved the user's control over the probe, leading to smoother translation and higher-quality images^17^. Both the original HRME and HF-HRME systems highlighted in this manuscript incorporate this probe holder. To improve the ratio of nuclear-to-cytoplasm signal, Tang et al. designed a confocal HRME with optical sectioning achieved by line scanning^18^. However, the system operated at 7–8 fps, which increased the likelihood of motion artifacts and reduced clinical feasibility. Future research could focus on increasing the frame rate of confocal HRME systems to reduce motion blur while maintaining high contrast. Finally, in this study, while the number of images that failed quality control was reduced, a small number of images were still manually eliminated, indicating the need for more robust, automated methods to advise the user on image quality. For example, other works have leveraged the nuclear segmentation output of the MTN to assess if sufficient nuclei are visible for diagnosis. This method accounts for out-of-contact frames but does not address the challenges with blurry images^19^.

New HRA practitioners require around 200 cases to attain proficiency in detecting anal HSIL^7^. The fast translation speed of the HF-HRME could allow novice clinicians to rapidly map large areas of the anal canal and target the detection of AIN 2+ areas that may have otherwise been missed. High-resolution mapping of nuclear morphology across large areas of tissue with HF-HRME was recently demonstrated in the cervix^20^. This process increases the effective field-of-view of the HF-HRME system and could potentially reduce the system’s dependence on HRA guidance for probe placement. Finally, high-resolution mapping of anal tissue with the HF-HRME could be used to direct resections and treatment.

This study shows that the HF-HRME significantly improves on the performance of the original HRME used in the previous pilot study. The high sensitivity and high specificity of the HF-HRME encourage the application of this system in anal cancer prevention strategies.

## Methods

### High frame rate high-resolution microendoscope

The HRME is a fiber-optic fluorescence microscope used to capture images of epithelial tissue stained with the fluorescent contrast agent proflavine. The HRME delivers blue excitation light (460 nm) from an LED through a coherent fiber bundle placed in contact with the epithelial tissue to be imaged. Resulting fluorescence is collected by the bundle and directed through a longpass filter to a camera. The HRME system used in the original pilot study used a monochrome CCD camera (CMLN-13S2M-CS; FLIR Systems Inc., Richmond, BC, Canada) with a frame rate of 15 fps; each pixel in the image corresponded to 0.78 µm at the tissue interface. When the fiber-optic bundle was translated across tissue at speeds exceeding 5 mm/s, images showed evidence of motion blur and often failed quality control. The maximal speed of translation of the original HRME bundle for effective imaging was significantly less than the speed of a pencil tip during writing, which ranges from 10 to 50 mm/s^21^. To address this challenge, we recently developed a high frame rate HRME (HF-HRME) that incorporates a high frame rate CMOS camera (BFS-U3-04S2M-CS; FLIR Systems Inc., Richmond, BC, Canada). Operating at a frame rate of 70 fps, the HF-HRME allows images to be captured at translation speeds of up to 15 mm/s without evidence of motion blur^17^. The HF-HRME is controlled by a LabVIEW user interface deployed on a high-performance laptop (MSI Creator Z17 A12UGST-049). The field of view and lateral spatial resolution of the HF-HRME are 790 µm and 4 µm, respectively, and each pixel in the image corresponds to 1.24 µm at the tissue interface. The goal of this study was to evaluate whether the HF-HRME would improve image quality and accuracy of AIN 2+ detection.

### Study participants

PLWH scheduled for routine surveillance at Thomas Street Health Center (Houston, Texas) were invited to participate. Patients were eligible to enroll if they were 18 years or older and had either a previous diagnosis of AIN of any grade or a positive cytology result (atypical squamous cells of undetermined significance or more severe) within the last 2 years. Individuals were excluded if they could not provide written informed consent or if they had: a platelet count less than 75,000 cells/mm^3^ and an absolute neutrophil count less than 1000 cells/mm^3^; a known permanent or irreversible bleeding disorder; or allergy or prior reaction to proflavine. The Institutional Review Board of Baylor College of Medicine and Affiliated Hospitals (Houston, Texas) approved this study (ID#:H-44616). The study was registered in ClinicalTrials.gov under the registration number NCT04563754 on 24/09/2020. All subjects provided written informed consent. All methods were performed in accordance with the study protocol and with the Declaration of Helsinki.

### Clinical procedures

Patients enrolled in the study first underwent standard of care HRA followed by HF-HRME imaging. During the HRA procedure, the patient was positioned on the examination table, and an anoscope was gently inserted to open the anal canal. The clinician used a standard colposcope (955 Colposcope, Seiler Medical, Missouri, USA) as the HRA imaging device to locate any lesions. Five percent acetic acid and Lugol’s iodine were used as contrast agents at the clinician’s discretion. The location of lesions (level and quadrant) and the corresponding HRA impression (HSIL or non-HSIL) were recorded. Following the HRA procedure, the clinician conducted HF-HRME imaging. Proflavine solution was first topically applied to the anal canal; the clinician then placed the fiber-optic probe of the HF-HRME in gentle contact with the tissue to capture videos from sites previously selected for biopsy. A live feed of the HF-HRME video was displayed to the clinician, who depressed a trigger to select a representative frame from the video to be analyzed with the real-time algorithm described below. The entire HF-HRME imaging session was recorded with the colposcope. All procedures were conducted by one clinical expert with over 10 years of experience in HRA. Biopsies were sent for histopathologic diagnosis with the following categories: benign, AIN 1, condyloma acuminatum, AIN 2, AIN 3, or cancer. Pathology was provided by a single study pathologist. P16 immunohistochemistry was used to validate results, with strong and diffuse positive p16 staining supporting AIN 2+ diagnoses.

### Deep learning model

All clinically selected HF-HRME images were scored in real-time using a previously developed deep learning model deployed on the study laptops^15^. The model consists of a MTN that performs nuclear segmentation and diagnostic classification. Nuclear segmentation is performed by the MTN’s encoder-decoder structure, which employs memory efficient operations to minimize the number of learnable parameters and allow deployment in resource-constrained computer systems such as portable laptops. The MTN’s diagnostic classification branch further reduced the feature maps generated by the nuclear segmentation encoder, culminating in a 64-element feature vector that was used by a final fully connected layer to predict the likelihood of AIN 2 +. The MTN was originally trained and validated for diagnosis of cervical intraepithelial neoplasia using HRME images from over 1600 women collected with the original HRME^15^. The previously validated model was used without any changes. Because HF-HRME and the original HRME have different spatial sampling, 1.24 and 0.78 um per pixel, respectively, HF-HRME images were resized (1.6 ×) to match the spatial sampling of the original HRME images. Scaled HF-HRME images were automatically cropped to isolate the fiber-optic probe and subdivided into quadrants; each passed through the MTN independently, and the corresponding probability of AIN 2+ was calculated. This process allowed the network to process HF-HRME images at full resolution. An HF-HRME image score was calculated by averaging the quadrant probabilities of AIN 2+. Here, we present the first application of the MTN to analyze HF-HRME images of the anal epithelium.

### Diagnostic evaluation metrics

Three experts retrospectively reviewed clinically selected HF-HRME images using the same quality control criteria as in the original pilot study. HF-HRME images were eliminated from the analysis set if the majority of experts concurred that over 50% of the field of view was blurry or out of contact^10^.

For sites that passed quality control, the real-time HF-HRME scores were used to compute the ROC curve and AUC for AIN 2+ diagnosis. The area under the precision-recall curve was also calculated. The sensitivity and specificity of HRA impression with respect to histopathology were calculated using HSIL impression as a positive HRA result. The operating point for the HF-HRME system was selected such that HRA impression and the HF-HRME system had equal sensitivity. The sensitivity and specificity of HRA and the HF-HRME were compared using McNemar’s test. The agreement between HRA impression and the HF-HRME was evaluated using Cohen’s κ. The specificities of the HF-HRME and the original HRME were compared at equal sensitivities using the Chi-square test.

### Identification of a feature shift

Compared to the original pilot study, this work took place in a new clinical setting, under the supervision of a different HRA practitioner, and with an HF-HRME system. Each of these factors impacted the data collected and could cause a shift in the distribution of the MTN’s features; the presence of study-specific clustering would indicate a significant feature distribution shift. To determine whether a shift occurred, we compiled the 64-element MTN feature vectors for all HF-HRME images in this study and HRME images in the original pilot study. A t-distributed stochastic neighbor embedding (t-SNE) visualization was used to visualize the clustering of these embeddings.

### Image perturbation study

We conducted an image perturbation study to investigate the relationship between changes in the imaging instrumentation and resulting MTN features between the original pilot study and this study. Images collected with the HF-HRME were processed to simulate the expected image that would have been collected if the original HRME had been used. The camera used in the HF-HRME (BFS-U3–04S2M-CS; FLIR Systems Inc., Richmond, BC, Canada) has a superior dynamic range of 74.35 and lower temporal dark noise of 3.8e- compared to the camera of the original HRME (CMLN-13S2M-CS; FLIR Systems Inc., Richmond, BC, Canada), which has a dynamic range of 57.85 and a temporal dark noise of 7.65e-. The Weber contrast of both devices was measured using a resolution target (Negative 1951 USAF Target). The noise floor was measured by acquiring an image of a blank target.

**Figure 5:**
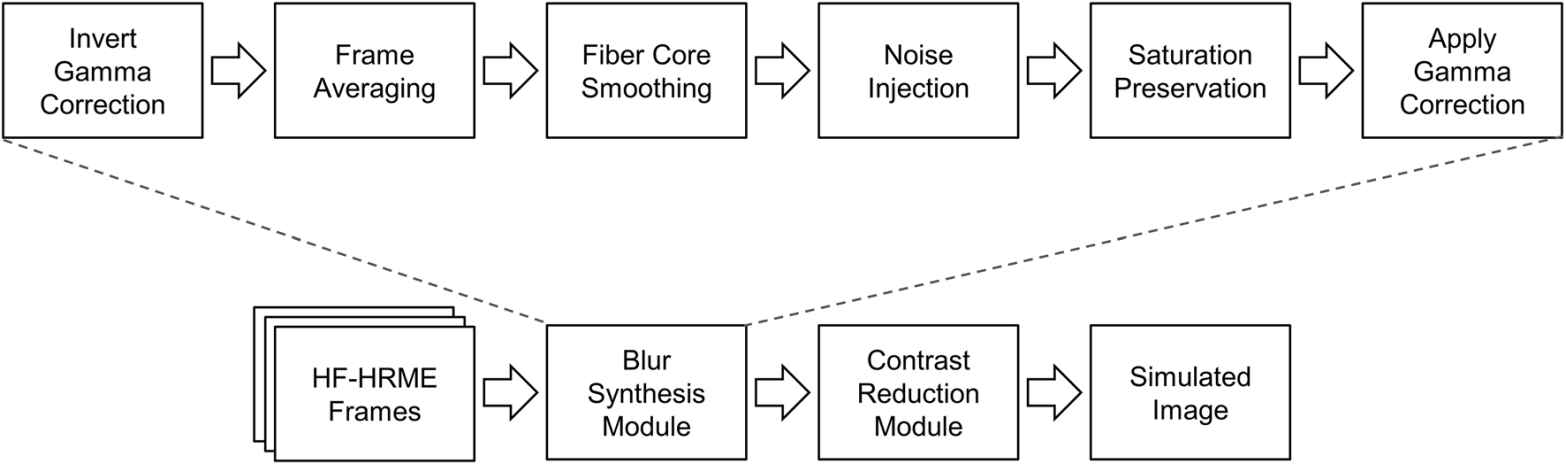
Image processing pipeline to process an image collected with the HF-HRME system and simulate the reduced frame rate and contrast of the HRME system used in the original pilot study. The image processing pipeline performs two main operations: blur synthesis and contrast reduction. *HF-HRME* high frame rate high-resolution microendoscope, *HRME* high-resolution microendoscope.

The image processing pipeline shown in Fig. 5 consists of an image blur synthesis module to simulate the expected image blur due to the reduced frame rate and a module to simulate the expected reduction in image contrast due to the increased noise floor of the original camera. The image blur synthesis module closely follows the methods of Rim et al. and aimed to simulate the blur of the HRME system used in the prior pilot study, which operated at 15 fps^22^. Briefly, the HF-HRME gamma correction was inverted to convert all frames into linear space. Then, the clinically selected HF-HRME frame and four preceding frames were averaged. Given that the HF-HRME exposure was 1.4 ms, averaging 5 frames generated a composite image with a 7 ms exposure, equivalent to a system running at 14 fps. However, averaging frames intensified the honeycomb pattern of the fiber-optic bundle, reduced image noise, and dampened saturation^22^. Thus, a small gaussian blur was applied to soften the honeycomb pattern, noise was injected into the image, and saturation areas present in the clinically selected image were preserved. Then, the gamma correction of the original HRME system was applied. Finally, the contrast reduction module employed color transfer to ensure that the composite image followed the grayscale distribution of the HRME images in the original pilot study^23^. Briefly, HF-HRME images and HRME images from the original pilot study were converted into CIELAB color space. The luminance channel was extracted and used in subsequent calculations. Each HF-HRME was mean normalized and divided by its standard deviation. The normalized HF-HRME images were multiplied and mean-shifted by the standard deviation and mean of the HRME images in the original pilot study, respectively.

To evaluate whether the image processing pipeline adjusted for differences in the instrumentation used in the two studies, we computed the average K–L divergence between the feature distributions for the HRME data collected in the original pilot study and the HF-HRME data collected in this study that were processed to simulate the reduction in frame rate and contrast. This value was compared to the average K–L divergence between the feature distributions for the HRME data collected in the original study and the actual HF-HRME data collected in this study. A reduction in average K–L divergence would indicate that the pipeline was successful in adjusting for instrumentation differences. An ablation study was performed to evaluate the impact of the four components within the blur synthesis module and of the contrast reduction module on K–L divergence. Transformation Method 1 employed all five components. Methods 2 to 5 removed one component each from the blur synthesis module; components were removed in the following order: gamma correction (Method 2), fiber core smoothing (Method 3), noise injection (Method 4), and saturation preservation (Method 5). Finally, Method 6 removed the contrast reduction module. The average scores, stratified by histopathologic diagnosis, of the transformation method with the lowest K–L divergence and the original pilot study were compared via T-Test. Finally, to estimate the impact of instrumentation improvements on diagnostic performance, we compared the area under the ROC curve when the MTN network was applied to the original HF-HRME data and HF-HRME data processed to simulate reductions in frame rate and contrast.

### Supplementary Information

Supplementary Video 1.

Supplementary Figures.

## Data Availability

The data that support the findings of this study are available from the corresponding author on reasonable request through a data-sharing agreement that provides for (i) a commitment to securing the data only for research purposes and not to identify any individual participant; (ii) a commitment to securing the data using appropriate computer technology; and (iii) a commitment to destroying or returning the data after analyses are completed.

## References

[1] Silverberg MJ (2012). Risk of anal cancer in HIV-infected and HIV-uninfected individuals in North America. Clin. Infect. Dis..

[2] de Martel C, Plummer M, Vignat J, Franceschi S (2017). Worldwide burden of cancer attributable to HPV by site, country and HPV type. Int. J. Cancer.

[3] Palefsky JM (2011). HPV vaccine against anal HPV infection and anal intraepithelial neoplasia. N. Engl. J. Med..

[4] Wilkin TJ (2018). A randomized, placebo-controlled trial of the quadrivalent human papillomavirus vaccine in human immunodeficiency virus-infected adults aged 27 years or older: AIDS Clinical Trials Group Protocol A5298. Clin. Infect. Dis..

[5] Clarke MA, Wentzensen N (2018). Strategies for screening and early detection of anal cancers: A narrative and systematic review and meta-analysis of cytology, HPV testing, and other biomarkers. Cancer Cytopathol..

[6] Lee JY (2022). Design of the anal cancer/HSIL outcomes research study (ANCHOR study): A randomized study to prevent anal cancer among persons living with HIV. Contemp. Clin. Trials.

[7] Richel O, Prins JM, de Vries HJC (2014). Screening for anal cancer precursors: What is the learning curve for high-resolution anoscopy?. AIDS.

[8] Silvera R (2021). The other side of screening: Predictors of treatment and follow-up for anal precancers in a large health system. AIDS.

[9] Palefsky JM (2022). Treatment of anal high-grade squamous intraepithelial lesions to prevent anal cancer. N. Engl. J. Med..

[10] Brenes D (2022). Automated in vivo high-resolution imaging to detect human papillomavirus-associated anal precancer in persons living with HIV. Clin. Transl. Gastroenterol..

[11] Castro DC, Walker I, Glocker B (2020). Causality matters in medical imaging. Nat. Commun..

[12] Melbye M, Sprøgel P (1991). Aetiological parallel between anal cancer and cervical cancer. Lancet.

[13] Scholefield JH (1989). Anal and cervical intraepithelial neoplasia: possible parallel. Lancet.

[14] Darragh TM, Winkler B (2011). Anal cancer and cervical cancer screening: key differences. Cancer Cytopathol..

[15] Brenes D (2022). Multi-task network for automated analysis of high-resolution endomicroscopy images to detect cervical precancer and cancer. Comput. Med. Imaging Graph..

[16] Wentzensen N (2021). Accuracy and efficiency of deep-learning-based automation of dual stain cytology in cervical cancer screening. JNCI J. Natl. Cancer Inst..

[17] Hunt B (2021). High frame rate video mosaicking microendoscope to image large regions of intact tissue with subcellular resolution. Biomed. Opt. Express.

[18] Tang Y, Carns J, Richards-Kortum RR (2017). Line-scanning confocal microendoscope for nuclear morphometry imaging. J. Biomed. Opt..

[19] Coole JB (2023). Multimodal optical imaging with real-time projection of cancer risk and biopsy guidance maps for early oral cancer diagnosis and treatment. J. Biomed. Opt..

[20] Coole JB (2022). Development of a multimodal mobile colposcope for real-time cervical cancer detection. Biomed. Opt. Express.

[21] Fujisawa Y, Okajima Y (2015). Characteristics of handwriting of people with cerebellar ataxia: Three-dimensional movement analysis of the pen tip, finger, and wrist. Phys. Ther..

[22] Rim, J. *et al.* Realistic blur synthesis for learning image deblurring. http://arxiv.org/abs/2202.08771 (2022).

[23] Reinhard E, Adhikhmin M, Gooch B, Shirley P (2001). Color transfer between images. IEEE Comput. Graph. Appl..

