# Supplementary material for: Deployment and assessment of a deep learning model for real-time detection of anal precancer with high frame rate high-resolution microendoscopy
Source: Sci Rep. 2023 Dec 14;13:22267. doi: 10.1038/s41598-023-49197-9 (PMC10721617; doi:10.1038/s41598-023-49197-9)
Supplement: Supplementary file 2 — Supplementary Figures. [file 41598_2023_49197_MOESM2_ESM.docx]

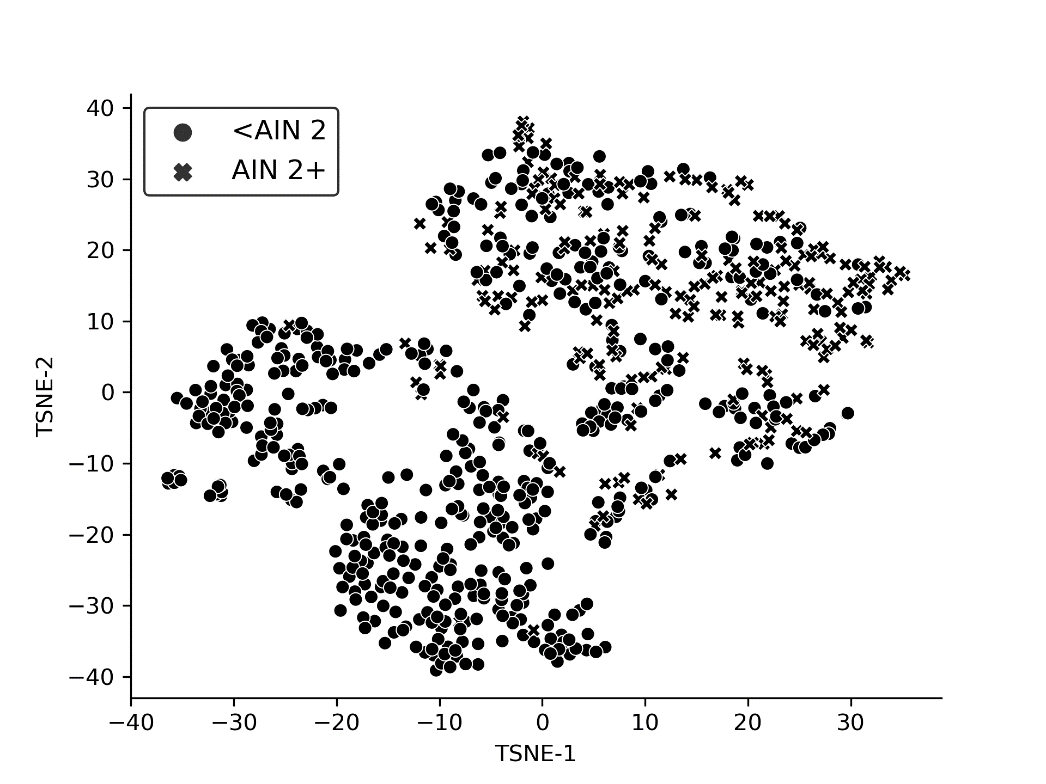


**Supplemental Figure 1.** t-SNE visualization of features generated by the MTN from images acquired with HF-HRME in the current study and with the original HRME in the pilot study. Each point corresponds to features from a single image quadrant and is labeled by pathology.

t-SNE, t-distributed stochastic neighbor embedding; MTN, Multi-Task Network; HF-HRME, high frame rate high-resolution microendoscope; HRME, high-resolution microendoscope.


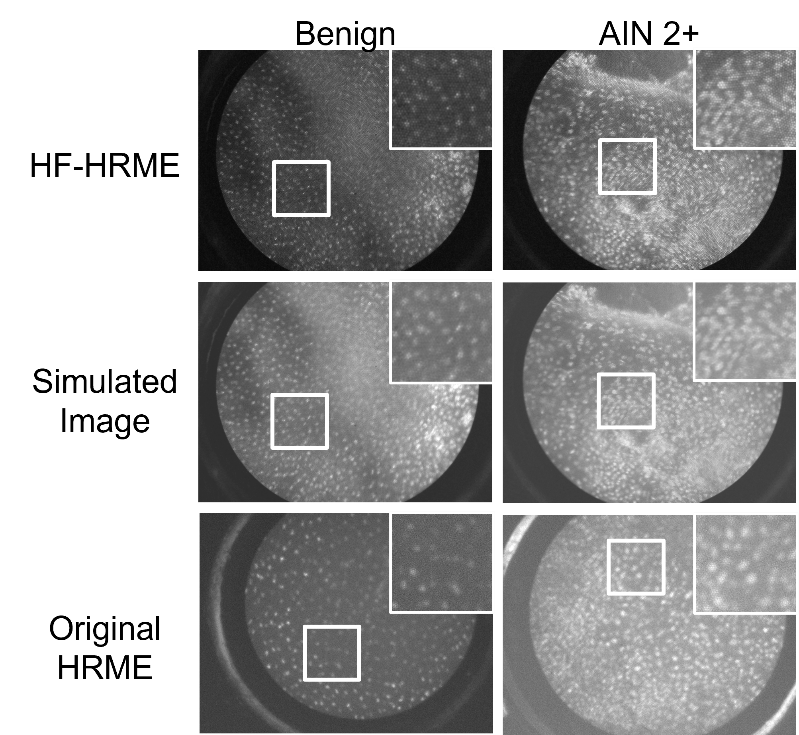


**Supplemental Figure 2.** Qualitative comparison of HF-HRME images, corresponding images processed to simulate their appearance if they were collected with the lower frame rate, lower contrast HRME system used in the original pilot study, and representative images collected in the original pilot study with the same histologic diagnosis.

HF-HRME, high frame rate high-resolution microendoscope; HRME, high-resolution microendoscope; AIN 2+, anal intraepithelial neoplasia grade 2 or more severe.
